# The illusion of orientation repulsion is weakened in a temporally more predictable visual target

**DOI:** 10.3758/s13414-025-03040-7

**Published:** 2025-03-24

**Authors:** Tomoya Nakamura, Ikuya Murakami

**Affiliations:** 1 Department of Psychology, The University of Tokyo, 7-3-1 Hongo, Bunkyo-ku, Tokyo, 113-0033 Japan; 2 Center for Brain Science, RIKEN, Saitama, Japan; 3 Japan Society for the Promotion of Science, Tokyo, Japan

**Keywords:** Temporal processing, Perceptual organization, Precueing

## Abstract

Anticipating the occurrence of future events enables our adaptive behavior by facilitating processing at various stages from perception to action. While the functional benefits of temporal expectation are well acknowledged, its phenomenological effects remain unknown. Focusing on the phenomenon of orientation repulsion, wherein a vertical target is perceived as tilted against surrounding stimuli, we examined how the size of the illusion varies with developing temporal expectation. In Experiment 1, a multimodal cue predicted impending target onset through its validity and rhythmicity. We found that repulsion decreased when the target appeared at or later than the moment predicted by the cue. In Experiment 2, rhythmic cues did not significantly influence repulsion without explicit instruction or subjective awareness of the cue–target contingency. In Experiment 3, a single cue was provided, and the target appeared after one of three foreperiods. The occurrence probability of the target was equalized across foreperiods to isolate the effect of the conditional probability given that the target had not yet occurred (hazard rate). Repulsion decreased as the hazard rate increased with the foreperiod. Heightened temporal expectations inevitably produce a phenomenological change in orientation repulsion by reducing perceptual latency, whereby a premature target representation that has not completely undergone contextual modulation is brought upon one’s perception.

## Introduction

Starting from the light projection patterns on the retina, the visual system performs intricate computations to construct a meaningfully organized perceptual representation. Contextual modulation is one of the most important characteristics that reflect these processes. In neurophysiology, modulations of neuronal responses by signals from outside the neuron’s receptive field are often argued in relation to adaptive functions, such as figure-ground segregation. For example, Lamme (1995) revealed that the visual responses of macaque V1 neurons to orientation-defined textures were enhanced several tens of milliseconds after the response onset, only if the texture inside the receptive field belonged to a figure. Such response enhancement had a longer delay as the recorded position moved farther from the figure-ground boundary (Lamme et al., 1999). As such, the process for neuronal contextual modulation is considered to be sluggish, allowing its dynamics to be tracked with neurophysiological methods.

In psychophysics, contextual modulation manifests as a phenomenological change in visual appearance, that is, what an object looks like. Indeed, basic features of a visual object, such as orientation, motion direction, depth, color, brightness, and size, are sometimes perceived as repulsive, that is, in the direction opposite to those in the spatial context surrounding the object, and sometimes perceived as attractive, that is, in the direction toward those in the context (Mély et al., 2018). Specifically, in the visual orientation domain, the orientation of a central object (called the target) appears to be tilted against the orientation of the surrounding stimuli (called the inducer), the phenomenon hereafter referred to as *orientation repulsion* (Gibson, 1937; Westheimer, 1990). The orientation repulsion size peaks when the orientation difference between the target and inducer is 10–20° (Clifford, 2014). Conversely, when it is 75–80°, the illusion reverses to attraction. Compared with orientation attraction, orientation repulsion is more robustly observed in various stimulus configurations, including line stimuli (Gibson, 1937; Westheimer, 1990), abutting sinusoidal gratings (e.g., Goddard et al., 2008; Mareschal & Clifford, 2012), and Gabor patches (Nakamura & Murakami, 2021, 2023; Solomon & Morgan, 2006, 2009). As for retinal eccentricity, repulsion occurs in both foveal (e.g., Goddard et al., 2008; Mareschal & Clifford, 2012) and peripheral (Nakamura & Murakami, 2021, 2023; Solomon et al., 2004) vision, with a stronger effect in the periphery (Over et al., 1972).

In line with the evolving nature of the neural representation for contextual modulation in orientation (e.g., Lamme, 1995; Lamme et al., 1999), psychophysical studies have demonstrated that the perception of orientation repulsion is also dynamically formed. Nakamura and Murakami (2021) quantified orientation repulsion when a four-dot masker surrounding the target disappeared together with the target offset and when the masker remained after the target offset, demonstrating that backward masking reduced the repulsion size. It was argued that the internal representation of the target orientation is initially faithful to the retinal image with no repulsion, evolving over time to get tilted away from the inducer’s orientation before the final outcome emerges on one’s conscious awareness; when backward masking interrupts this evolution, the target’s premature representation becomes accessible by a process responsible for one’s conscious awareness, resulting in a weaker repulsion being consciously experienced. In a similar vein, Nakamura and Murakami (2023) found that the repulsion was weaker when the target was preceded by a flanker stimulus providing temporal facilitation than when these stimuli appeared simultaneously without temporal facilitation. It was suggested that the preceding flanker accelerated the target’s emergence into one’s conscious awareness at an earlier point in time before contextual modulation has been completed.

In the present study, we aimed to investigate whether these effects on visual appearance are driven only by *external* signals provided just before (i.e., flanker) or after (i.e., masker) the target, or whether similar effects are also produced by the observer’s *internal* model about time. Specifically, we examined whether orientation repulsion is also altered by the expectation as to when a target is more likely to occur in the current environmental setup. Below, we review classical studies demonstrating how such temporal expectations shorten the processing time for visual stimuli and argue how temporal expectation could influence the temporal evolution of contextual modulation.

### Concept of temporal expectation

In our daily lives, recognizing the temporal structure of events in our environment is crucial. For instance, a change in the color of a traffic light from green to yellow is a predictive cue that the light will soon turn red, prompting drivers to slow down. To perceive and respond to dynamic situations adequately, we adaptively refer to the temporal contingencies or regularities inherent in the environment, thereby developing “temporal expectations” of upcoming events.

In laboratory settings, temporal expectation is typically manipulated through the probability of stimulus onset as a function of time. Here, we mention three well-established experimental paradigms for manipulating temporal expectations (Nobre & van Ede, 2018; Seibold et al., 2023). The first paradigm is temporal orienting (e.g., Coull & Nobre, 1998; Miniussi et al., 1999; see also Denison et al., 2017). Analogous to the Posner paradigm for spatial orienting (Posner, 1980), a target is typically presented at one of two distinct time intervals. A symbolic pre-cue indicates the interval in which the target is more likely to appear. With an informative cue (e.g., 80% cue validity), the reaction time (RT) is shorter when the target appears in the cued interval than in the uncued interval. However, this effect of cue validity diminishes when the target appears during the second interval. The reason for the diminishment is given by the concept of the “hazard rate,” that is, the probability of an event occurring at a specific point in time given that the event has not occurred by then (Coull, 2009; Nobre et al., 2007). If the target does not appear in the first interval, it will certainly appear in the second interval (i.e., the hazard rate has become the unity). Thus, it will be advantageous to re-prepare for the second interval regardless of cue validity. Indeed, if a certain proportion of trials are catch trials with no target presentation, the cue validity effect is observed for the second interval as well (e.g., Correa et al., 2004, Correa, Lupiáñez, et al., 2006).

The second paradigm is rhythmic entrainment (e.g., Jones et al., 2002; Rohenkohl et al., 2012). Prior to the target presentation, cues are repeated in a certain rhythm. Reactions are quicker for targets that appear in phase with the cue rhythm than for those that appear out of phase (Breska & Deouell, 2014; Sanabria et al., 2011). Furthermore, the RT for targets embedded in a regular rhythm is shorter than that for the targets embedded in an irregular rhythmic pattern (Cravo et al., 2013; Rohenkohl et al., 2012). According to the dynamic attending theory (see Jones, 2010), rhythmic stimuli automatically entrain neural oscillations by aligning their phases and periods, enhancing the perception of events synchronized with the rhythm (e.g., Mathewson et al., 2010; Ronconi & Melcher, 2017). Indeed, temporal expectations shaped by rhythmic stimuli often trigger the entrainment of slow-wave delta-band oscillations (e.g., Cravo et al., 2013; Lakatos et al., 2008; Will & Berg, 2007). In any case, the performance enhancement through rhythmic entrainment indicates that we utilize the temporal predictability given by external rhythms, even in the absence of explicit instructions (but see Elbaz & Yeshurun, 2020).

The third paradigm is variable foreperiod (e.g., Han & Proctor, 2022; Los et al., 2001; Niemi & Näätänen, 1981). A single cue precedes a target by a variable length of time called the foreperiod, and RT typically decreases as the foreperiod increases. This can be explained by the higher hazard rates associated with longer foreperiods (Coull, 2009; Nobre et al., 2007), and indeed, this relationship is attenuated when the hazard rate is held constant using non-aging foreperiod distributions, such as exponential and geometric distributions (Näätänen, 1971; see also Vangkilde et al., 2012). By contrast, an alternative interpretation arises from the sequential effect (e.g., Los et al., 2001; Los & Van Den Heuvel, 2001), in which RT also depends on the foreperiod of one trial if it is longer than the current foreperiod. This trace conditioning account claims that the conditioning for periods shorter than the current foreperiod is extinguished owing to the absence of a target, and the conditioning for a period equally long to the current foreperiod is reinforced, while the conditioning for longer periods remains unchanged. This asymmetry in conditioning strength mimics the asymmetry in the sequential effect (see Los et al., 2001). However, recent studies have demonstrated a dissociation between the neural loci for the foreperiod and sequential effects (but see Los et al., 2014). For example, transcranial magnetic stimulation (TMS) to right dorsolateral prefrontal cortex (rDLPFC) attenuates the foreperiod effect but not the sequential effect (Vallesi, Shallice et al., 2007); the surgical removal of brain tumors in rDLPFC also selectively attenuates the foreperiod effect (Vallesi, Mussoni et al., 2007; see also Vallesi et al., 2009); the sequential effect emerges ontogenetically earlier than the foreperiod effect (Vallesi & Shallice, 2007).

### The processing stages influenced by temporal expectation

Although it has traditionally been considered that temporal expectation primarily influences motor processes such as response preparation and execution (e.g., Coull & Nobre, 1998; Hasbroucq et al., 1999; Los et al., 2001; Mattes & Ulrich, 1997; Näätänen, 1971), recent studies have demonstrated that it also influences earlier processes. For example, Bausenhart et al. (2006) used the psychological refractory period (PRP) paradigm (see, e.g., Pashler, 1984) to explore the pre-motor effects of temporal expectations. In the standard PRP paradigm, participants quickly respond to two successive stimuli (S1 and S2). Owing to a bottleneck at the response-selection stage, the RT to S2 increases as the stimulus-onset asynchrony (SOA) between S1 and S2 decreases. Bausenhart et al. (2006) found that the temporal uncertainty of S1 lengthened RT to S2 only when the SOA was short, indicating a slower response selection process. Additional support for the effect of temporal expectations on pre-motor processes has emerged from event-related potential (ERP) studies. Hackley et al. (2007) divided the time course from stimulus presentation to response into three stages: stimulus to N2-posterior-contralateral (N2pc), N2pc to lateralized readiness potential (LRP), and LRP to RT. They found that the length of the foreperiod influenced only the second interval, indicating an improvement in post-perceptual/pre-motor processes, such as response selection (see also Müller-Gethmann et al., 2003).

**Fig. 1:**
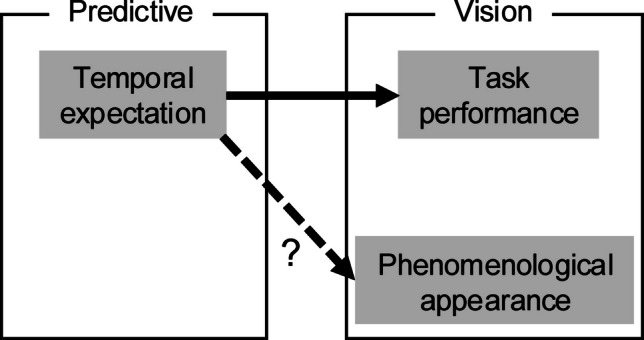
The relationship of the concepts relevant to our study. The solid arrow indicates a relatively established effect, whereas the broken arrow accompanied by a question mark indicates the influence that we examined

Studies examining performance measures other than RT have shown that temporal expectations influence even visual processing. Temporal uncertainty elevates the perceptual threshold (Lasley & Cohn, 1981; Westheimer & Ley, 1996) and reduces perceptual discriminability (Rolke & Hofmann, 2007; see also Correa et al., 2005; Rohenkohl et al., 2012). In line with these psychophysical observations, electrophysiological studies have shown that temporal expectations modulate the amplitudes of early ERP components such as N1 (e.g., Doherty et al., 2005; Lange, 2009; Lange et al., 2003) and P1 (Correa, Lupiáñez, et al., 2006). In summary, the prevailing view is that temporal expectations influence various stages of the processing hierarchy, enhancing performance in diverse tasks. However, it remains unknown whether temporal expectations can alter visual appearance (Fig. 1). In previous studies, experimental manipulations of detectability/discriminability were not necessarily accompanied by changes in appearance (Prinzmetal et al., 2008; Schneider & Komlos, 2008; but see Carrasco et al., 2004). To observe the effect on appearance, it is essential to quantify a phenomenologically malleable illusion instead of threshold and discriminability.

### The present study

The present study assessed the visual appearance of orientation repulsion in the presence of variability in temporal expectations. It has been argued that orientation repulsion is closely related to the iso-orientation surround suppression in the early visual cortex (especially V1), where the neural response to stimuli in the receptive field center is suppressed when the surroundings have a similar orientation (Goddard et al., 2008; Levitt & Lund, 2002). If temporal expectation can influence the appearance of visual features tightly connected to such hardwired low-level visual processing, orientation repulsion will change with developing temporal expectation. By contrast, if temporal expectations are not allowed to touch compelling phenomenology, orientation repulsion will not change. We tested which scenario is the case.

In Experiment 1, we examined a situation in which temporal cues accurately predicted target onset time. To achieve this, we merged temporal orienting with rhythmic entrainment. The repulsion was reduced for a target presented at the predicted moment. To identify the pivotal factors responsible for this effect, we conducted two following experiments. In Experiment 2, we focused on the impact of rhythmic entrainment, and found no evidence of a reduction in repulsion caused solely by rhythmicity. In Experiment 3, we focused on the effect of foreperiod length to further examine the contribution of the hazard rate and found that repulsion decreased with an increase in the foreperiod, even though the occurrence probability remained constant across foreperiods.

In addition, to ensure that temporal expectation was properly manipulated in our procedure, RTs for orientation discrimination were measured as an index of task performance in additional sessions in which no orientation illusion was induced.

## Experiment 1

### Method

#### Participants

Eleven naïve adults (eight men and three women; aged 20–26 years) participated. All had normal or corrected-to-normal visual acuity and reported no hearing deficiencies. The sample size was determined using G-Power 3.1 (Faul et al., 2009). To detect an expected effect size (Cohen’s ${d}_{z}$ = 1.19) based on a previous study that revealed a reduction in orientation repulsion in a similar configuration (Nakamura & Murakami, 2023), a paired t-test with a power of .95 and a significance level of .05 necessitated a minimum of 12 participants. The study initially included 12 participants, but the data for one participant had to be excluded afterward because of naïvety, resulting in a final power of .94. However, the patterns of all the statistical results did not differ before and after the exclusion.

#### Apparatus

The experimental procedure was controlled using the MATLAB R2019b programming environment (MathWorks, Natick, MA, USA) and Psychophysics Toolbox Version 3 (Brainard, 1997; Kleiner et al., 2007; Pelli, 1997), running on a Dell Precision computer with Ubuntu 18.04.5. All visual stimuli were presented against a gray background (31 cd/m^2^) on a cathode ray tube (CRT) monitor (Mitsubishi Electric RDF223H) with a refresh rate of 60 Hz and a spatial resolution of 1,600 × 1,200 pixels. To eliminate potential references for orientation judgment, the edges of the monitor were obscured with a round occluder made of black cardboard. In a darkroom, the participants viewed the visual stimuli while resting their heads on a chin and head rest 57 cm from the monitor. All auditory stimuli were delivered binaurally through portable headphones (Audio-Technica ATH-AR3).

#### Stimuli

**Fig. 2:**
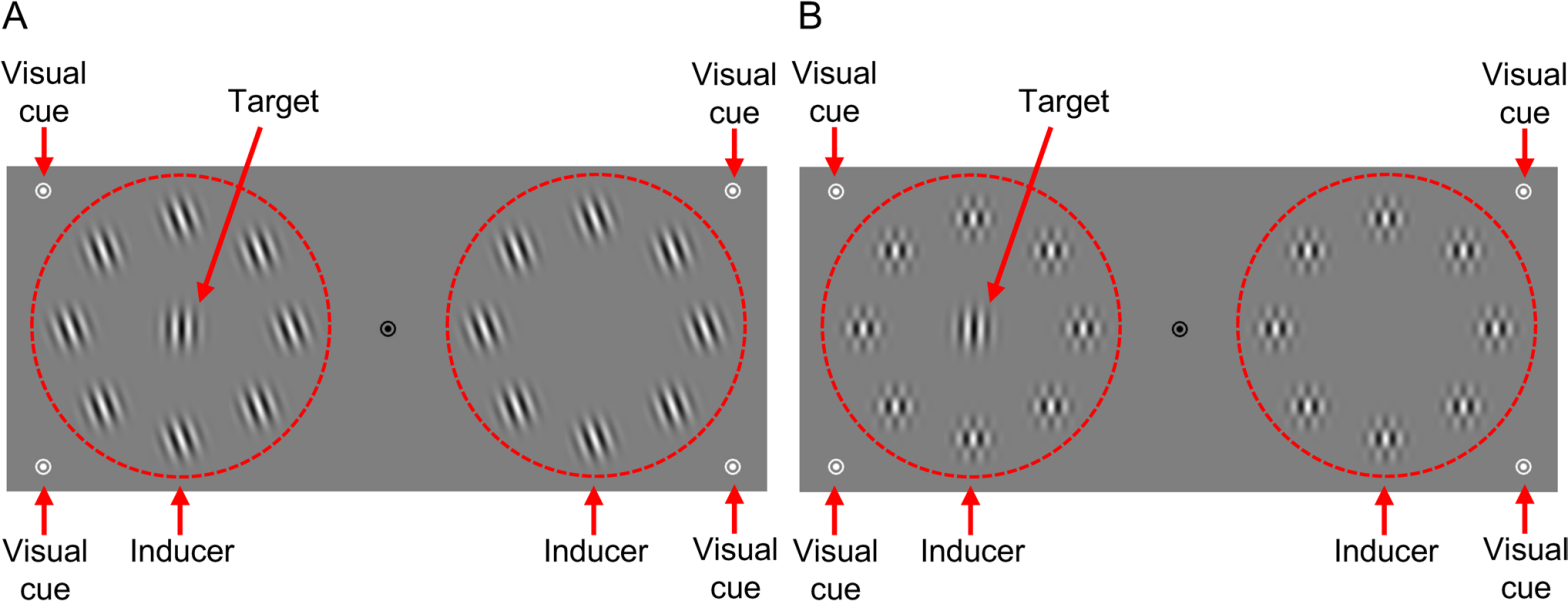
Stimulus configurations. (**A**) Main sessions for the measurement of orientation repulsion. (**B**) Additional session for the measurement of reaction time. The broken circles and arrows are only for illustrative purposes and were not shown to the participants

The stimulus configurations were virtually the same as those used in previous studies that demonstrated the evolving nature of orientation repulsion with a sufficient illusion size at its maximum (Nakamura & Murakami, 2021, 2023). Throughout each experimental block, the participants were requested to maintain fixation on a black bullseye (0.8° × 0.8°) at the center of the monitor (Fig. 2A). Gabor patches (spatial frequency: 2.0 cpd; Gaussian standard deviation: 0.42 deg; Michelson contrast: 99%) were used as both the target and inducer stimuli for orientation repulsion. The target Gabor patch was flashed at 7.5° to the left or right of the bullseye. Around both potential target locations, eight Gabor patches were evenly spaced on an imaginary circle with a radius of 4.0°. These patches were tilted either 20° or −20° relative to the vertical (positive degrees denoting a clockwise orientation). The phase of the inducer patches was consistent within a trial but randomized from trial to trial. The phase of the target patch was randomized independently of the inducer phase. The range of the target orientation was individually calibrated before the main experiment, as described below. Given that audition typically outperforms vision in temporal processing (e.g., Repp & Penel, 2002; Shipley, 1964) and that cross-modal cues enhance the effect of temporal expectation (ten Oever et al., 2014), we presented visual and auditory cues synchronously. Their physical synchronicity was confirmed using an oscilloscope. The visual cue consisted of white (62 cd/m^2^) bullseyes (0.8° × 0.8° each) positioned at the four vertices of a virtual rectangle (25° wide × 10° high) centered on the fixation point. The auditory cue was a 960 Hz pure tone presented at a comfortable sound pressure level. The amplitude during the initial and final quarters of the tone duration was tapered using a raised-cosine filter.

#### Procedure

##### Measurement of orientation repulsion (main sessions)

**Fig 3:**
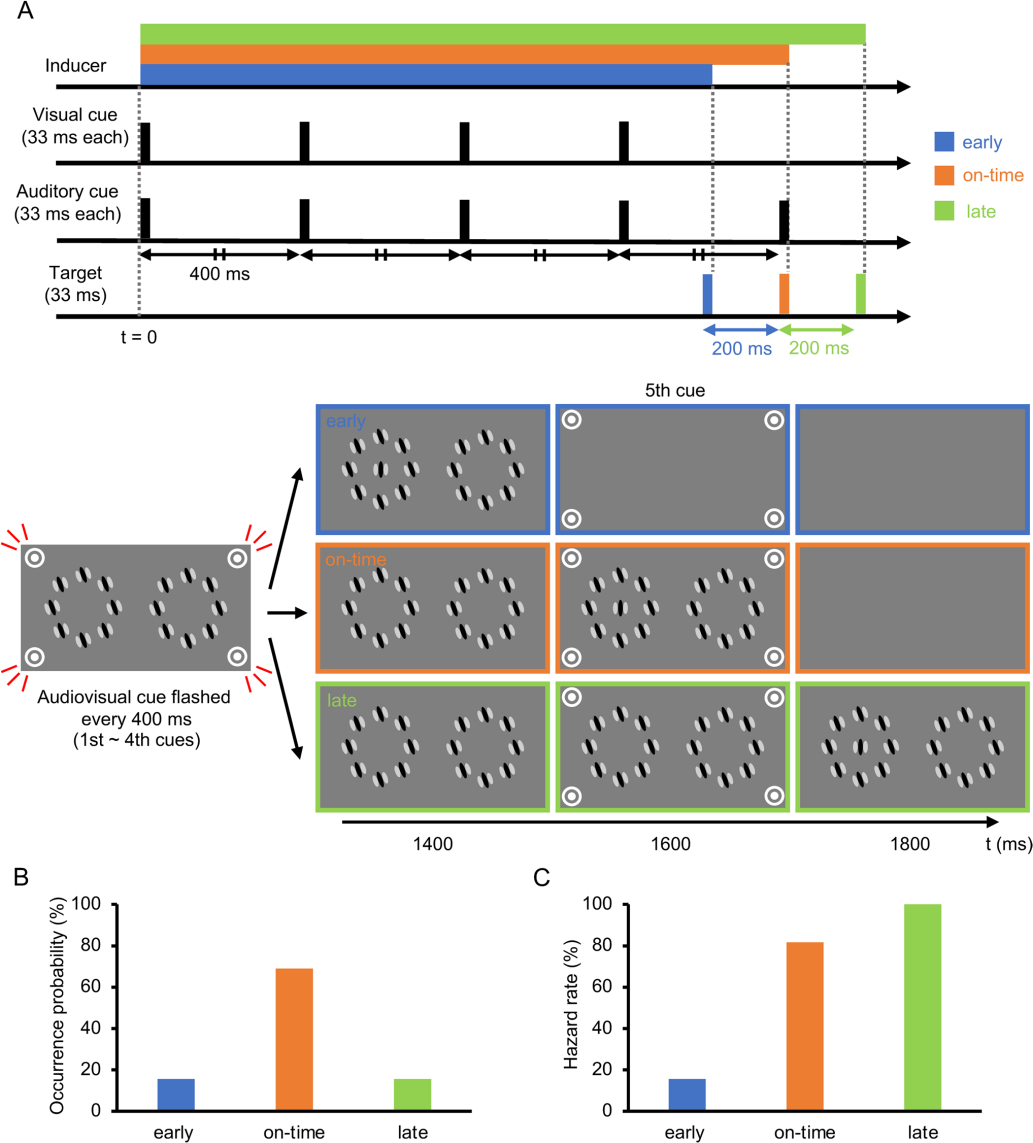
Procedure for Experiment 1. (**A**) Time course of one trial in the early (blue/dark gray), on-time (orange/middle gray), and late (green/light gray) conditions. (**B**) Occurrence probability for each condition. (**C**) Hazard rate under each condition

Each trial began at the onset of the inducer. Subsequently, the audiovisual cues were delivered four times at intervals of 400 ms, each lasting 33 ms (Fig. 3A), followed by the fifth cue, which consisted only of the auditory cue. In 68.9% of trials (62 out of 90 trials in each block), the target coincided with the fifth auditory cue. The participants were instructed beforehand to attend to the time of the fifth cue as the target was expected to appear at this particular time with the highest probability. These trials were categorized as the “on-time” condition. In one-half of the remaining trials (15.6%), the target appeared 200 ms earlier than the fifth cue (“early” condition). In the other half (15.6%), the target appeared 200 ms later (“late” condition). Under any of these conditions, the inducer was turned off concurrently with the target offset to equate the inducer duration during a period that was effective for repulsion (i.e., within approximately 100 ms around the target presentation; see Mareschal & Clifford, 2012). Each participant was asked to indicate via a keypress whether the target was perceived as tilted clockwise (CW) or counterclockwise (CCW) relative to the vertical orientation. Each response triggered the next trial after a 1,000-ms interval. As such, we maximized the environmental cues for participants to predict the moment of the impending target by deliberately fixing the time course such that the target moment was technically predictable not only from the audiovisual cues but also from the inducer onset (i.e., the target would most likely appear 1,600 ms after the inducer appeared) and the moment of the preceding response (i.e., the target would most likely appear 2,600 ms after the last response was made).

Each session consisted of two blocks, each containing 90 trials. Within each session, the participants performed 124 trials (2 inducer orientations × 2 target locations × 31 target orientations) in the on-time condition and 28 trials (2 inducer orientations × 2 target locations × 7 target orientations) in each of the early and late conditions in a random order. In total, 14 sessions were completed over 2 days, with an adequate rest interval between successive blocks.

##### Determination of target orientation range (calibration session)

Prior to the abovementioned main sessions, a calibration session was conducted to determine the range of target orientations to be tested. For each participant, orientation repulsion size (denoted as ${OR}_{pre}$) in the “on-time” condition was roughly predetermined using the Psi method (Kontsevich & Tyler, 1999). We randomly interleaved four staircases (2 inducer orientations × 2 target locations), each of which ended in 30 trials.

$$O{R}_{pre}=\frac{PS{V}_{CW,right}+PS{V}_{CW,left}-PS{V}_{CCW,right}-PS{V}_{CCW,left}}{4}$$

where PSV indicates the point of subjective verticality, and the suffixes denote the inducer orientation and target location. In the main sessions, the target orientation was ranged within ${OR}_{pre}$ ± 6° for the CW-inducer condition and − ${OR}_{pre}$ ± 6° for the CCW-inducer condition. Within each range, the levels of target orientation in the early and late conditions were set at 2° intervals (comprising seven levels), whereas those in the on-time condition were set at 0.4° intervals (comprising 31 levels) to ensure a higher occurrence probability because of the larger number of trials (Fig. 3B).

Using the method of constant stimuli, we plotted the percentage of CW responses against the target orientation and fitted it with a logistic function using the maximum likelihood method implemented in the Palamedes Toolbox (Kingdom & Prins, 2016). The slope was constrained between the CW- and CCW-inducer conditions because there were no theoretical grounds for predicting any differences in orientation discriminability between these two conditions. The lapse rate was fixed at .02 (see Wichmann & Hill, 2001). The orientation repulsion size was quantified as half the distance between the PSVs newly derived from the best-fit psychometric functions for the CW- and CCW-inducer conditions. The discrimination threshold for orientation was quantified as the difference between the PSV and the target orientation corresponding to the 75% CW response rate.

##### Measurement of reaction time (RT; additional session)

**Fig 4:**
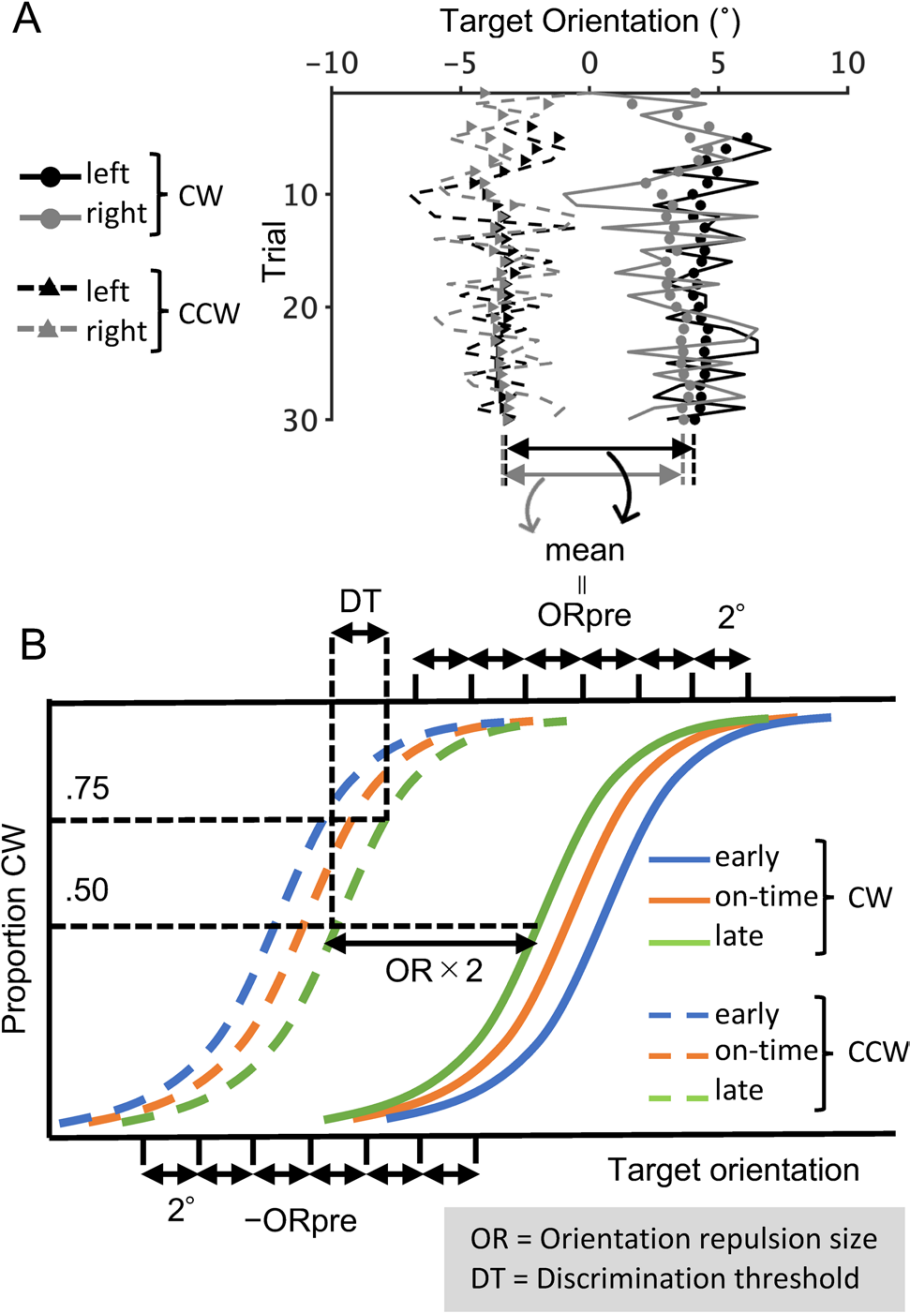
Protocols for calibrating the range of target orientation based on data in the calibration session and for estimating orientation repulsion size in the main sessions. (**A**) Four staircases during calibration session. The lines indicate the orientation of the target presented in each trial and the markers indicate the best estimate of the point of subjective verticality (PSV) after each trial. The mean difference in PSV between the clockwise (CW) and counter-clockwise (CCW) conditions (${OR}_{pre}$) was used to determine the center of the target orientation range in the main sessions. Data from one representative participant are shown. (**B**) Derivation of psychometric function from main session data. In the main sessions, the target orientation was chosen from ${OR}_{pre}$ ± 6° for the CW condition and ${-OR}_{pre}$ ± 6° for the CCW condition. OR and DT indicate the orientation repulsion size and discrimination threshold, respectively (those under the late condition are exemplified here)

To explore the classical effect of temporal expectations on perceptual performance, the RT for an orientation discrimination task was measured in an additional session composed of two blocks of 90 trials, which included 124 trials (2 target locations × 2 target orientations × 31 repetitions) in the on-time condition and 28 trials (2 target locations × 2 target orientations × 7 repetitions) each in the early and late conditions. The stimuli and task in this separate session were consistent with those in the main sessions, except for the following. Each inducer patch was substituted with a plaid pattern having a luminance profile corresponding to the pixel-wise luminance average of two Gabor patches tilted by 20° and −20° (Fig. 2B). Note that, because the two opposing orientations were superimposed, the inducer patch did not induce an orientation illusion. The target was tilted either CW or CCW from the vertical by 1.6 times the discrimination threshold in the on-time condition during the main session (Fig. 4B). The participants were instructed to respond as quickly and accurately as possible during this additional session.

#### Statistical analyses

Initially, data normality was tested using a Shapiro–Wilk test for each dependent variable. In cases where the normality was violated, we performed a nonparametric Friedman test. Otherwise, we performed a repeated-measures analysis of variance (rm-ANOVA) with the degrees of freedom adjusted using Greenhouse-Geisser’s $\varepsilon$, calculated based on Mendoza’s sphericity test. Whenever pairwise comparisons were performed, *p*-values were corrected using Holm’s (1979) method. Furthermore, to take into account the within-subject uncertainty of the dependent variables, we calculated the confidence intervals for the effect sizes by resampling data from the best-fit psychometric function using the bootstrap method.

For the RT data in Experiment 1, we also conducted a nonparametric permutation test because uneven numbers of trials across conditions could bias the test statistics (Ernst, 2004; see also Rohenkohl et al., 2014). In the permutation test, we randomly shuffled the condition labels of the trials within each participant and computed the test statistics (*F* and *t*). A total of 10,000 permutations yielded null distributions for the test statistics. The probability that the test statistic in the null distribution is more extreme than the empirical value is reported as ${p}_{perm}$.

### Results and discussion

In the main sessions, a vertical target was surrounded by a tilted inducer, and the target could appear just on time, 200 ms earlier, or 200 ms later, relative to the moment predicted by the temporal cues, where the predicted moment means the moment at which the participants were instructed to orient their attention because the target would appear with the highest probability.

**Fig 5:**
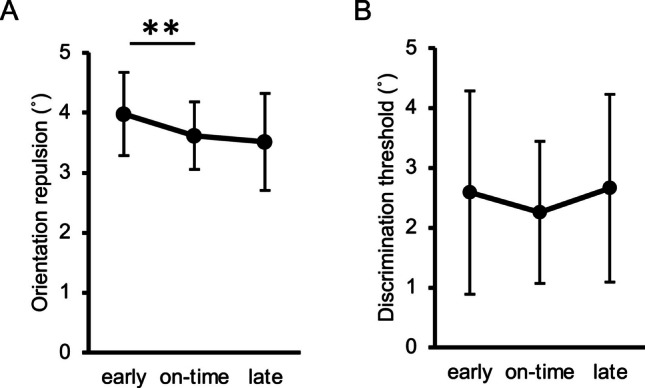
Results of the main sessions in Experiment 1. (**A**) Inter-participant mean of orientation repulsion size. (**B**) Inter-participant mean of discrimination threshold of orientation. Error bars indicate 95% confidence intervals. ** *p* <. 01

Since orientation repulsion size violated normality (*W* = .900, *p* = .007), we conducted a Friedman test, finding that the main effect of target timing on orientation repulsion size (Fig. 5A) was significant, ${\chi }^{2}$(2) = 9.45, *p* = .009, *W* = .430, 95% CI = [.058, .603]. Pairwise comparisons using Wilcoxon signed-rank tests revealed that repulsion in the early condition was significantly stronger than in the on-time condition,* V* = 62, *p* = .020, *r* = .777, 95% CI = [.268, .885], but there was no significant difference between the on-time and late conditions, *V* = 43, *p* = .413, *r* = .268, 95% CI = [−.188, .509]. Therefore, the repulsion depended on the temporal predictability informed by the cues, such that it was greater when the target appeared earlier but not later than expected. The pattern of repulsion sizes appeared to follow the hazard rate (Coull, 2009; Nobre et al., 2007), which was 15.6%, 81.6%, and 100% in the early, on-time, and late conditions, respectively (Figure 3C). However, this pattern was not inconsistent with the objective probability distribution of the target’s occurrence (Figure 3B), which should have been rejected if and only if the difference in repulsion between the early and late conditions reached significance; but in fact, it did not, *V* = 57, *p* = .064, *r* = .643, 95% CI = [.215, .858].

Prior to the experimental sessions, the participants were verbally instructed about the probability distribution of the target occurrence. In real life, however, temporal expectations should be formed as a result of statistical learning through experiences. To examine whether any progress with respect to such statistical learning was observed while each participant was spending 2 days to complete the experiment, we analyzed the data separately for each day and compared them between the 2 days. We focused on the pair of conditions that exhibited a significant reduction in repulsion in the previous analysis (early vs. on-time conditions). To compensate for the reduction in statistical power caused by halving the number of trials, we merged the data across target and inducer orientations, calculating the “proportion opposite,” i.e., the proportion of responses made opposite to the inducer orientation. A previous study has confirmed that this simplified index is consistent with the orientation repulsion size determined by a more conventional sigmoidal fitting procedure (see Nakamura & Murakami, 2021). We conducted a two-way rm-ANOVA on the “proportion opposite,” which did not significantly violate normality (*W* = .984, *p* = .792), finding that the main effect of target timing, *F*(1, 10) = 17.12, *p* = .002, ${\eta }_{p}^{2}$ = .631, but neither the main effect of day, *F*(1, 10) = 0.35, *p* = .566, ${\eta }_{p}^{2}$ = .034, nor their interaction, *F*(1, 10) = 0.11, *p* = .750, ${\eta }_{p}^{2}$ = .011, was significant. In other words, orientation repulsion was reduced in the on-time condition compared to the early condition, with similar reduction sizes between the 2 days. This finding suggests that the participants had already internalized the probability distribution by the beginning of the main sessions on the first day, and also displays a degree of repeatability, although we could not find a significant correlation in the reduction of repulsion between the 2 days (*r* = −.092, *p* = .788), presumably due to the limited data size.

Since discrimination threshold (Fig. 5B) also violated normality (*W* = .896, *p* = .006), we conducted a Friedman test. In contrast to the repulsion data, the discrimination threshold did not significantly differ across the conditions, ${\chi }^{2}$(2) = 1.64, *p* = .441, *W* = .075, 95% CI = [.000, .256], meaning that there was no indication that temporal expectation could improve orientation discriminability.

**Fig. 6:**
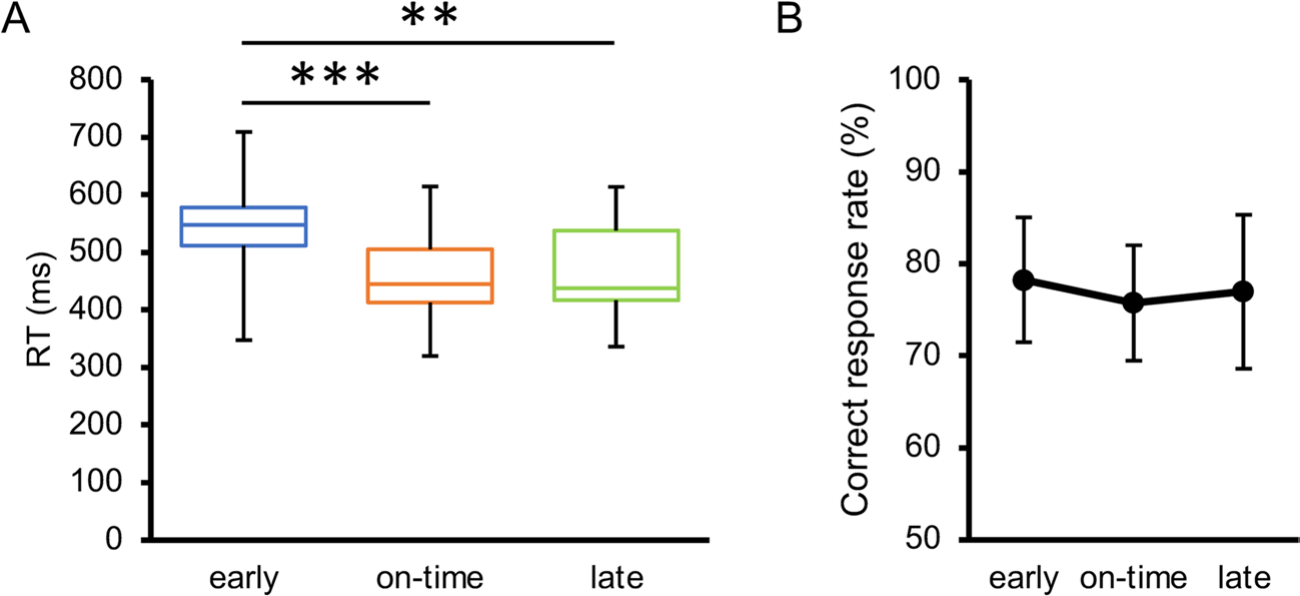
Results of the additional session in Experiment 1. (**A**) Inter-participant mean of reaction time (RT) in the additional session. (**B**) Inter-participant mean of correct response rate in the additional session. Error bars indicate 95% confidence intervals. ** *p* <. 01, *** *p* < .001

In the additional session, we measured the RTs in the early, on-time, and late conditions. The participants were instructed to discriminate the orientation of a target surrounded by plaid patterns as quickly and accurately as possible. For each participant and condition, the RTs for trials with correct responses were averaged, and the RTs greater than 3 SD from the mean were excluded as outliers. Neither the within-subject mean RT (*W* = .960, *p* = .226) nor the correct response rate (*W* = .397, *p* = .967) violated normality. The main effect of target timing on RT (Fig. 6A) was highly significant, *F*(1.4, 14.1) = 13.76, *p* = .001, ${\eta }_{p}^{2}$ = .579, 95% CI = [.221, .621], ${p}_{perm}$ < .001. Pairwise comparisons revealed that it took more time to judge the target orientation in the early condition than in the on-time and late conditions, *t*(10) = 6.07, *p* < .001, ${d}_{z}$ = 1.83, 95% CI = [0.85, 2.12], ${p}_{perm}$< .001, *t*(10) = 3.44, *p* = .013, ${d}_{z}$ = 1.04, 95% CI = [0.34, 1.23], ${p}_{perm}$ = .002. RT did not significantly differ between the on-time and late conditions, *t*(10) = 0.31, *p* = .766, ${d}_{z}$ = 0.09, 95% CI = [−0.45, 0.20], ${p}_{perm}$ = .618. Since the correct response rate (Fig. 6B) did not significantly differ across the conditions, *F*(1.8, 18.3) = 0.19, *p* = .813, ${\eta }_{p}^{2}$ = .018, 95% CI = [.001, .159], ${p}_{perm}$ = .679, the difference in RT could not be attributed to a speed-accuracy tradeoff. The RT results can also be explained by the development of hazard rates over time (Coull, 2009; Nobre et al., 2007). If the target did not appear at the early moment, the hazard rate (Fig. 3C) radically increased (from 15.6% to 81.6%); however, if the target did not appear even at the on-time moment, it increased to a lesser extent (from 81.6% to 100%). Consequently, RT decreased in the on-time condition compared to the early condition but did not further improve in the late condition.

## Experiment 2

In Experiment 1, the participants were explicitly instructed to attend to the specific moment at which the target would most likely be presented – the “on-time” moment. It is possible that the participants were automatically entrained to the temporal frequency of the cues owing to their rhythmic nature (2.5 Hz). This entrainment may have elevated the preparatory state at the on-time moment. In Experiment 2, we focused on the effect of rhythmic entrainment on orientation repulsion without any instructions about temporal predictability that might be informed by cues.

### Method

#### Measurement of orientation repulsion (main sessions)

**Fig. 7:**
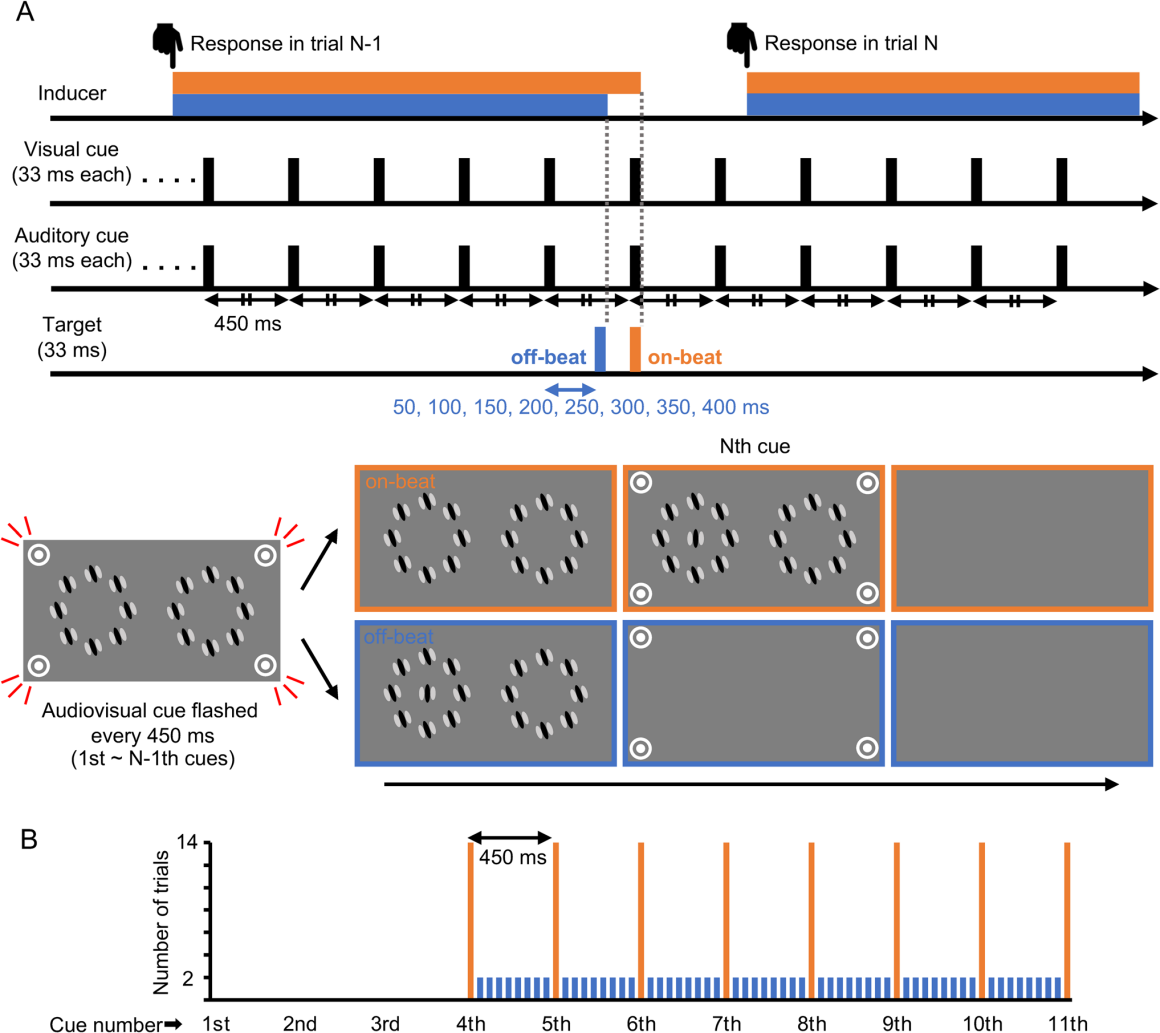
Procedure for Experiment 2. (**A**) Time course of one trial in the on-beat (orange/light gray) and off-beat (blue/dark gray) conditions. The response in the previous trial triggered the onset of the inducer in the subsequent trial. (**B**) Distribution of target timing relative to the cue timing. The cue number on the abscissa counts from the inducer onset, whereas the ordinate indicates the number of trials in one session

Another 11 naïve adults (nine men and two women; aged 20–23 years) participated. The apparatus, stimuli, and task were the same as in Experiment 1; however, unlike Experiment 1, in which five cues were presented in sequence in each trial, the cues in Experiment 2 were repeated at consistent intervals of 450 ms throughout each experimental session. The presentation of either a CW- or CCW-inducer signaled the beginning of each trial, and the target, lasting 33 ms, appeared at some time point between the fourth and 11th cues (cue numbering began from the onset of the inducer in each trial). In half of the trials, the target was presented in synchrony with one of the cues (referred to as the “on-beat” condition). In the remaining half, the target was presented out of phase with the cues (referred to as the “off-beat” condition); the SOA between the target and the cue immediately preceding it was set to 50, 100, 150, 200, 250, 300, 350, or 400 ms (Fig. 7A). It is important to note that, while the probabilities of the on-beat and off-beat conditions were equal, the distribution of target timing in relation to cue timing was non-uniform (see Fig. 7B). Nevertheless, as the inducer in each trial appeared immediately after the response in the preceding trial, it lacked temporal contingency with the ongoing cue stream. Consequently, while the inducer onset roughly signaled the imminent presentation of the target, the participants could not predict the moment of the target’s occurrence unless they employed a complicated strategy of counting cues from the inducer onset. To further prevent the participants from attending to the cue stream, they were explicitly instructed to ignore the rhythmic aspect and concentrate solely on discerning the orientation of the target. Moreover, introspective reports after the experiment verified that no temporal contingency was detected between the cue and target. At the moment the target disappeared, the inducer always disappeared together, and the cue stream continued during each intertrial interval demarcated by the disappearance of the current inducer and the appearance of a new inducer for the next trial.

Each session consisted of four blocks, each containing 56 trials. Within each session, the participants performed 112 trials (2 inducer orientations × 2 target locations × 7 target orientations × 4 repetitions) in each of the on-beat and off-beat conditions in a random order (the distribution of target timing in a session is illustrated in Fig. 7B). In total, four sessions were completed in 1 day.

#### Determination of target orientation range (calibration session)

Before the main sessions, the range of the target orientation was determined following the same protocol as in Experiment 1. In this calibration session, no cue was provided to eliminate any cue–target contingency, and the SOA between the inducer and target was randomly chosen from a flat distribution ranging from 1.35 to 2.40 s. The target and inducer disappeared simultaneously, as in the main sessions.

#### Measurement of RT (additional session)

In an additional session, the RT for the orientation discrimination task was measured in two blocks, each having 56 trials. In this session, the participants performed 56 trials (2 target locations × 2 target orientations × 14 repetitions) in each of the on-beat and off-beat conditions in a random order. The stimuli and task were the same as those in the additional session of Experiment 1, except that the target tilt was 2.1 times the discrimination threshold derived from the main session data. The distribution of target timing in each session was the same as that in the main sessions (see Fig. 7B).

#### Statistical analyses

For the statistical analyses, we conducted a Wilcoxon signed-rank test as a nonparametric test, and a paired t-test as a parametric test.

### Results and discussion

**Fig. 8:**
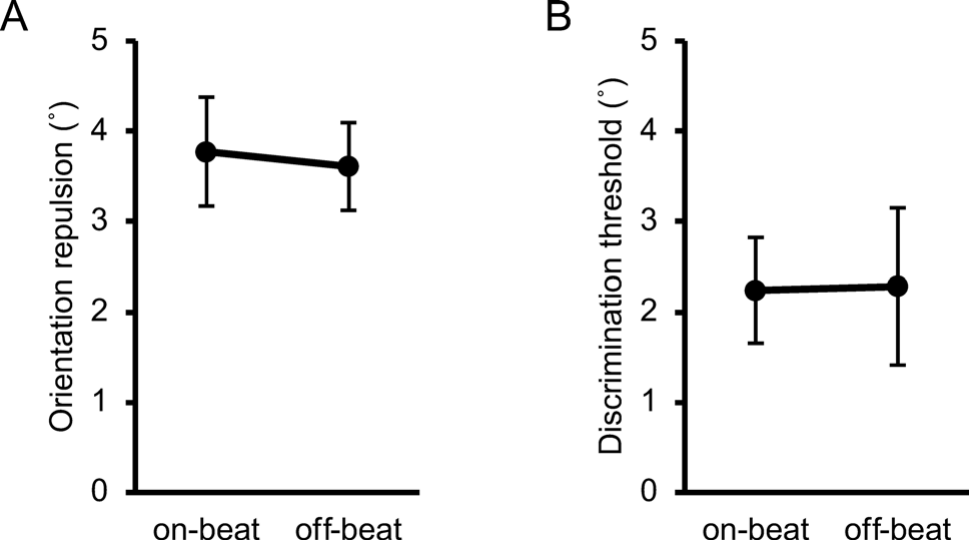
Results of the main sessions in Experiment 2. (**A**) Inter-participant mean of orientation repulsion size. (**B**) Inter-participant mean of discrimination threshold of orientation. Error bars indicate 95% confidence intervals

The orientation repulsion size did not violate normality (*W* = .983, *p* = .959), but the discrimination threshold did (*W* = .869, *p* = .009). Neither the orientation repulsion size (Fig. 8A) nor the discrimination threshold (Fig. 8B) significantly differed between the on-beat and off-beat conditions, *t*(10) = 0.96, *p* = .360, ${d}_{z}$ = 0.29, 95% CI = [−0.10, 0.59]; *V* = 40, *p* = .577, *r* = .188, 95% CI = [−.321, .456]. Therefore, the cue rhythm per se did not effectively influence the appearance of orientation repulsion. As confirmed by post-experimental introspective reports, all participants in Experiment 2 were unaware of temporal predictability given by the rhythm. This was not the case for the participants in Experiment 1, who were verbally instructed to attend to the moment at which the target would most likely appear. Unlike Experiment 2, the number of cues preceding the target was fixed in Experiment 1, which also helped the participants know which cue to focus on. Therefore, we suggest that the rhythm’s predictability can be effective only when participants are aware of the predictability and can be prepared for the impending target.

**Fig. 9:**
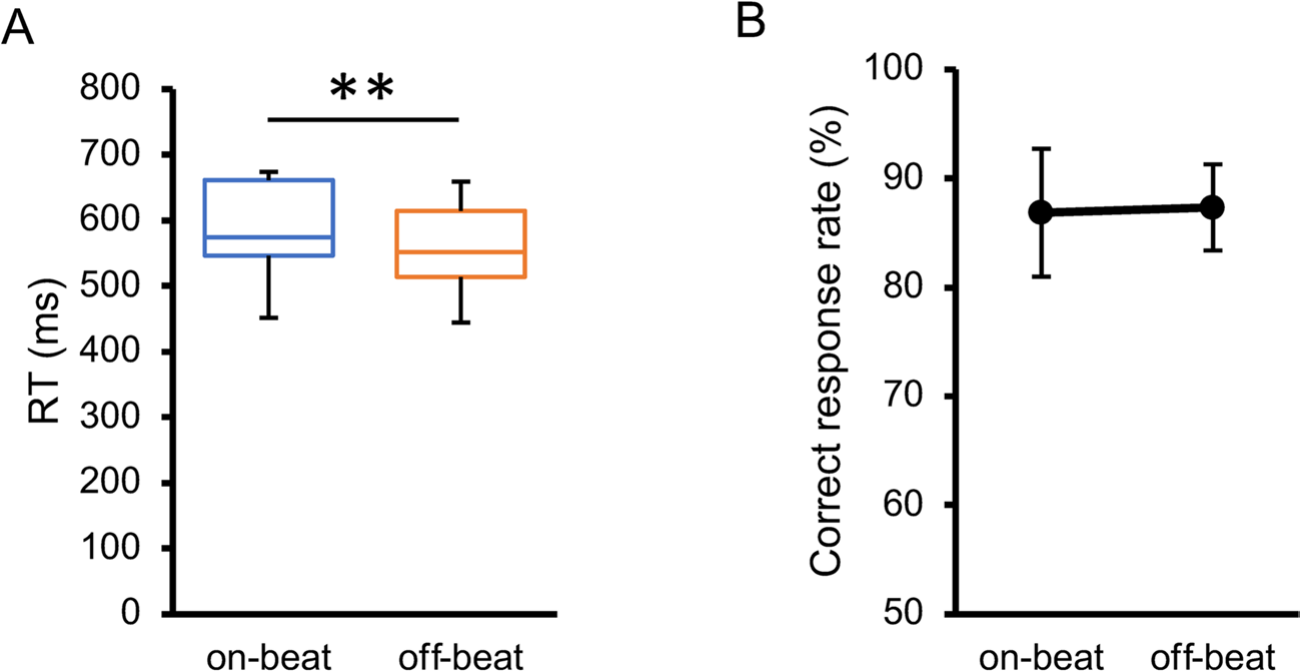
Results of the additional session in Experiment 2. (**A**) Inter-participant mean of reaction time (RT) in the additional session. (**B**) Inter-participant mean of correct response rate in the additional session. Error bars indicate 95% confidence intervals. ** *p* <. 01

As for the additional session, neither the within-subject mean RT (*W* = .917, *p* = .064) nor the correct response rate (*W* = .992, *p* = .999) violated normality. The off-beat condition yielded significantly quicker RTs than the on-beat condition, *t*(10) = 3.37, *p* = .007, ${d}_{z}$ = 1.02, 95% CI = [0.29, 1.45] (Fig. 9A). The correct response rate (Fig. 9B) did not significantly differ across conditions, *t*(10) = 0.38, *p* = .711, ${d}_{z}$ = 0.12, 95% CI = [−0.48, 0.63]; therefore, the difference in RT could not be attributed to a speed-accuracy tradeoff. This difference in RTs contrasts with previous studies indicating that rhythmic events facilitate RTs for on-beat targets (Rohenkohl et al., 2012; Sanabria et al., 2011). We can only speculate about the source of this discrepancy; the instructed strategy to ignore the cue sequence might have resulted in some suppression of the processing of the target in phase with the rhythm (Bauer et al., 2015; see also Spaak et al., 2014); and the presence of the simultaneous cue – not presented in previous investigations – may also have automatically distracted the participants’ attention from the target (Prasad et al., 2021; see also Ishigami et al., 2009). This speculation is statistically supported by the fact that RTs in the *on-beat* condition in Experiment 2 were significantly slower than those in the *on-time* condition in Experiment 1, *t*(20) = 3.42, *p* = .003, ${d}_{s}$ = 0.44, which indicates generally slower RTs in Experiment 2 than in Experiment 1, even though the target appeared in sync with the rhythmic cue in both conditions.

## Experiment 3

In Experiment 1, neither orientation repulsion nor RT exhibited significant variations between the on-time and late conditions despite the difference in occurrence probability. This lack of difference in RT can be explained by considering the hazard rate (Coull, 2009; Nobre et al., 2007). However, the influence of the hazard rate on orientation repulsion remains unknown. To distinguish the effect of the hazard rate from the effect of temporal orienting based on cue validity, the classical paradigm of the variable foreperiod (Los et al., 2001; Niemi & Näätänen, 1981) was employed in Experiment 3. A target was equally likely to appear at one of multiple moments after a temporal cue; nevertheless, the hazard rate monotonically increased as time elapsed after the cue.

### Method

#### Measurement of orientation repulsion (main sessions)

**Fig. 10:**
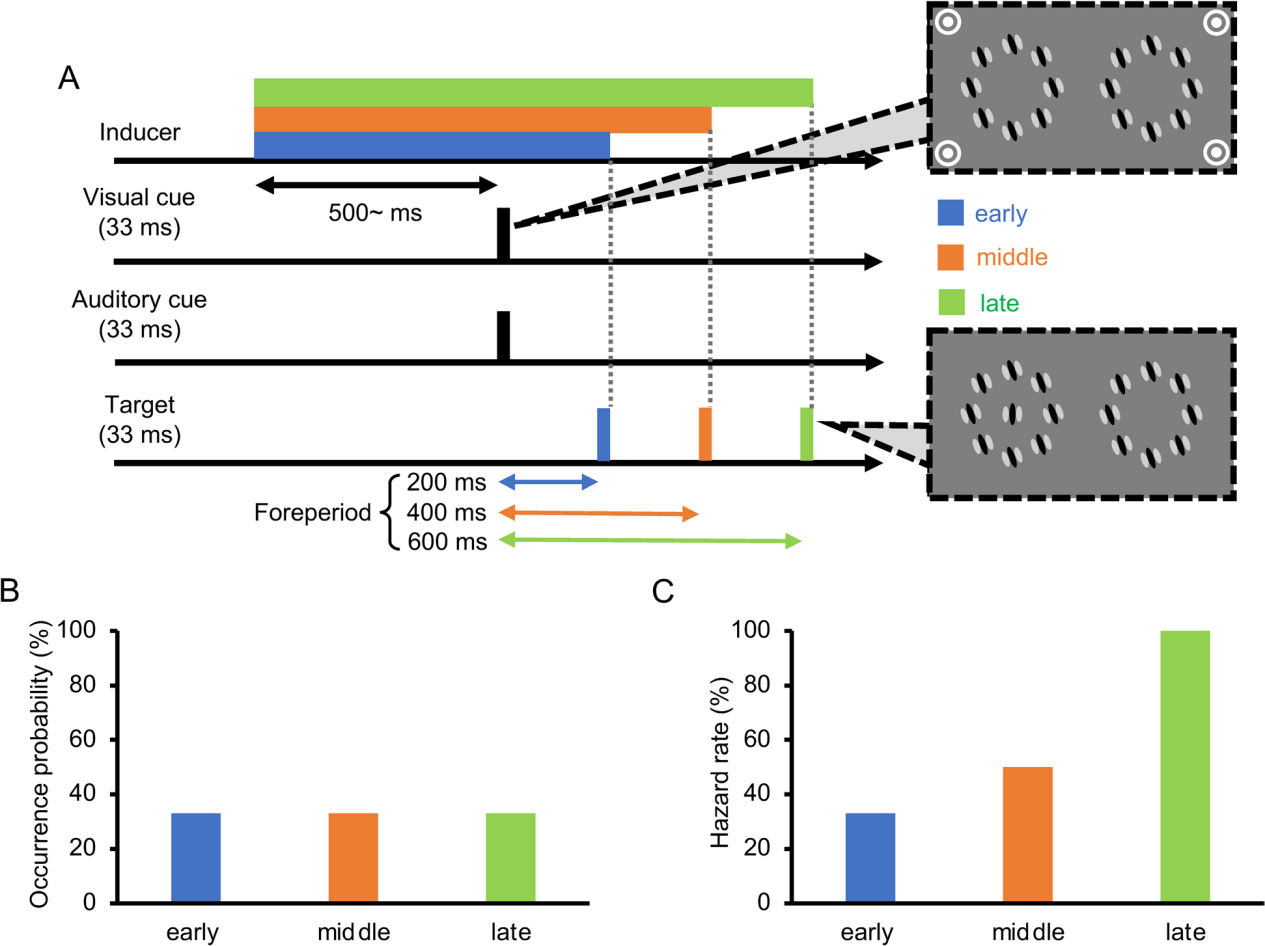
Procedure for Experiment 3. (**A**) Time course of one trial. The foreperiod between the cue and target was 200, 400, and 600 ms in the early (blue/dark gray), middle (orange/middle gray), and late (green/light gray) conditions, respectively. (**B**) Occurrence probability for each condition. (**C**) Hazard rate under each condition

All the participants in Experiment 2 participated in Experiment 3 a few days later. All the participants maintained their naïvety as debriefing was not planned between the experiments. The apparatus, stimuli, and task were the same as those used in Experiment 1. The presentation of an inducer signaled the initiation of each trial (Fig. 10A). Importantly, the cue was presented only once during each trial. In Experiment 1, the target was temporally cued not only by the audiovisual cues but also by the inducer onset (i.e., the target would most likely appear 1,600 ms after the inducer appeared) and the moment of the preceding response (i.e., the target would most likely appear 2,600 ms after the last response was made). In Experiment 3, we neutralized the latter two components, isolating the first predictive component solely based on the audiovisual cue. Specifically, the inducer–cue SOA was set at 500 + X ms, where X was randomly drawn from an exponential distribution (a type of “non-aging” distribution rendering the hazard rate constant against elapsed time) with a mean of 1,000 ms: such deliberate introduction of uncertainty has been demonstrated to heighten the impact of the most proximate temporal cue, the audiovisual cue in our case (Bausenhart et al., 2010; Müller-Gethmann et al., 2003). The target was presented after a cue–target SOA, hereafter referred to as the foreperiod, which was 200, 400, and 600 ms in the early, middle, and late conditions, respectively. The occurrence probability was constant across the three foreperiods (Fig. 10B), whereas the hazard rate increased with foreperiod length (Fig. 10C). Each response triggered the next trial after a 1,000-ms interval.

Each session consisted of two blocks, each containing 84 trials. Within each session, the participants performed 56 trials (2 inducer orientations × 2 target locations × 7 target orientations × 2 repetitions) in each of the early, middle, and late conditions in a random order. In total, seven sessions were completed in one day.

#### Measurement of RT (additional session)

An additional RT session was identical to that in Experiment 2, except for the following: it comprised two blocks, each having 84 trials. In this session, the participants performed 56 trials (2 target locations × 2 target orientations × 14 repetitions) in each of the early, middle, and late conditions in a random order. As in Experiment 2, the target tilt was 2.1 times the discrimination threshold derived from the main session data.

#### Statistical analyses

Since all dependent variables in the main sessions (orientation repulsion: *W* = .976, *p* = .656; discrimination threshold: *W* = .958, *p* = .220) and the additional session (within-subject mean RT: *W* = .950, *p* = .116; correct response rate: *W* = .974, *p* = .606) did not violate the normality assumption, we conducted rm-ANOVAs. Whenever pairwise comparisons were performed, *p*-values were corrected using Holm’s (1979) method.

### Results and discussion

**Fig. 11:**
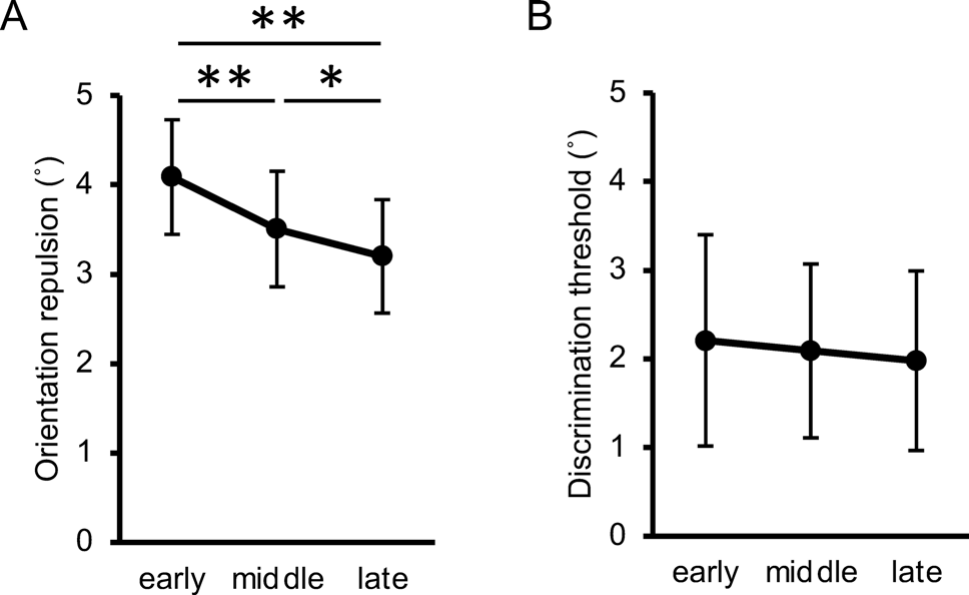
Results of the main sessions in Experiment 3. (**A**) Inter-participant mean of orientation repulsion size. (**B**) Inter-participant mean of discrimination threshold of orientation. Error bars indicate 95% confidence intervals. * *p* <. 05, ** *p* < .01

The main effect of the foreperiod on orientation repulsion (Fig. 11A) was significant, *F*(1.5, 15.3) = 22.09, *p* < .001, ${\eta }_{p}^{2}$ = .688, 95% CI = [.357, .731]. Pairwise comparisons revealed that repulsion in the early condition was significantly greater than that in the middle and late conditions, *t*(10) = 4.76, *p* = .002, ${d}_{z}$ = 1.44, 95% CI = [0.47, 1.80],* t*(10) = 5.26, *p* = .001, ${d}_{z}$ = 1.59, 95% CI = [0.87, 2.12]. Moreover, repulsion in the middle condition was significantly greater than that in the late condition, *t*(10) = 2.81, *p* = .019, ${d}_{z}$ = 0.85, 95% CI = [0.11, 1.15]. In contrast to repulsion data, the discrimination threshold (Fig. 11B) did not significantly differ across the conditions, *F*(1.6, 15.8) = 2.18, *p* = .153, ${\eta }_{p}^{2}$ = .179, 95% CI = [.006, .294].

**Fig. 12:**
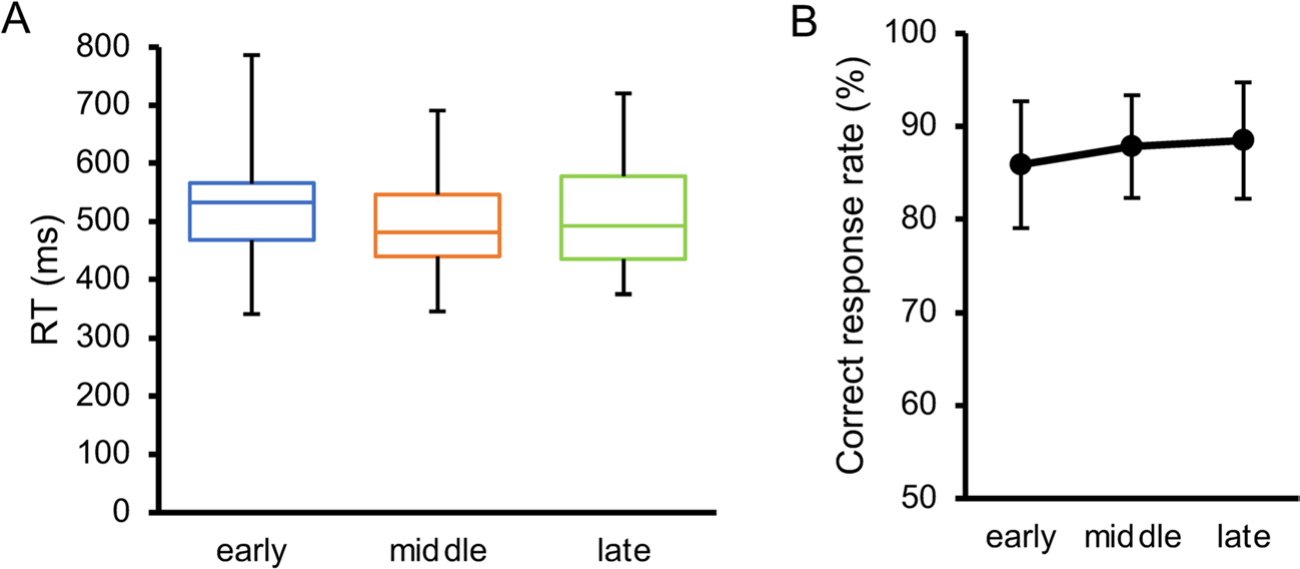
Results of the additional session in Experiment 3. (**A**) Inter-participant mean of reaction time (RT). (**B**) Inter-participant mean of correct response rate. Error bars indicate 95% confidence intervals

Although the main effect of foreperiod on RT (Fig. 12A) was not significant, *F*(1.7, 16.5) = 2.70, *p* = .105, ${\eta}_{p}^{2}$ = .212, 95% CI = [.033, .344], the numerical trend was comparable to that observed in Experiment 1 in Steinborn et al. (2008), where RT was assessed with exactly the same foreperiod range as in our study. Specifically, RT decreased as the foreperiod increased up to 400 ms but did not continue to decrease further. As we determined our sample size for the repulsion data, more participants may be necessary to detect the effect of the foreperiod on RT. In addition, the correct response rate (Fig. 12B) did not significantly differ across the conditions, *F*(1.3, 13.2) = 0.41, *p* = .588, ${\eta }_{p}^{2}$ = .040, 95% CI = [.002, .215].

## General discussion

### Summary of the results

Orientation repulsion was smaller when the target appeared at or later than the moment to which attention was drawn by the temporal cue’s validity and its rhythmic structure (Experiment 1). Nonetheless, the rhythmic structure of cues alone failed to modify repulsion, at least when the participants were not explicitly informed or aware of the temporal contingency between the cue and target (Experiment 2). Furthermore, when the foreperiod between a single temporal cue and target was manipulated within an experimental block, repulsion decreased as the foreperiod increased (Experiment 3).

### Psychological mechanisms

In the temporal expectation examined in our study, the temporal distribution of event occurrences must be quickly learned and stored within a process capable of handling statistics in the perisecond range and updating the hazard rates over the time course of a single trial. Considering the temporal tuning of visual modules (Fritsche et al., 2020; Gauthier et al., 2012), it is more plausible that such learning occurs at the post-perceptual level, where statistics in the perisecond time domain are manageable (Herbst et al., 2018; Janssen & Shadlen, 2005; see also Coull, 2009). Additionally, the results of Experiment 2 demonstrated the importance of awareness of temporal predictability, further ruling out low-level sources as the origin of the effects observed in our study. In contrast, orientation repulsion is well-established as a perceptual phenomenon in nature (e.g., Clifford, 2014) and is known to be immune to post-perceptual biases (Patten & Clifford, 2015). Since we employed a two-alternative forced-choice paradigm that required responses immediately following target onset, the involvement of mnemonic processing should be minimal compared to the method of adjustment. This suggests that the observed reduction in repulsion likely occurred at the perceptual stage. Therefore, our findings – that temporal expectations regarding the target’s occurrence weaken orientation repulsion (Experiments 1 and 3) – indicate that the temporal expectation, possibly formed by computations at the post-perceptual stage, acts on the perceptual stage (see, e.g., Correa, Lupiáñez, et al., 2006; Rolke & Hofmann, 2007).

How does the post-perceptual information of temporal expectations influence representation at the perceptual stage? Rolke and Hofmann (2007) found that temporal certainty improves shape discrimination under masking, suggesting that temporal expectation hastens the onset of the accumulation of target information, and this temporal advantage helps the target escape masking. In line with this “early onset view,” the onset of centroparietal positivity (CPP), an ERP component predominantly associated with the accumulation of perceptual information, is expedited by temporal expectations (Devine et al., 2019; van den Brink et al., 2021). Furthermore, the “early onset view” can also account for other effects caused by temporal expectation, including the dilation of perceived duration (Grondin & Rammsayer, 2003), the acceleration of perceptual latency (Seifried et al., 2010), and the shift in the onset of the speed-accuracy tradeoff function (Bausenhart et al., 2010).

By contrast, model-based studies indicate that temporal expectations accelerate the rate of the accumulation process rather than hastening its onset (Rohenkohl et al., 2012; Vangkilde et al., 2012, 2013; but see Jepma et al., 2012; van den Brink et al., 2021). For example, Rohenkohl et al. (2012) measured RT and correct orientation-discrimination rates for a target Gabor patch presented within regular and irregular streams of distractors. The psychometric and chronometric functions were simultaneously fitted with a drift-diffusion model, which posits that a perceptual decision is made once the accumulated information reaches a certain criterion (see Palmer et al., 2005). The model fit was successful when a faster accumulation rate was assumed under the regular condition than under the irregular condition, but not when a difference in decision onset time was assumed between conditions. This “faster accumulation view” accounts for the psychophysical finding that temporal expectations reduce the just noticeable difference in a temporal order judgment task (Bausenhart et al., 2008; Correa, Sanabria et al., 2006).

Our results agree with both “early onset view” and “fast accumulation view,” if it is assumed that the internal representation related to orientation repulsion evolves over time (Nakamura & Murakami, 2021) and is accumulated before reaching one’s awareness. Consider a scenario in which a vertical target is surrounded by a CW-tilted inducer. The initial accumulation following the target onset reflects the retinal input (vertical orientation). However, as contextual modulation takes effect over time, the internal representation of the target orientation gradually evolves into a CCW tilt. If the target onset is predicted more accurately by a temporal cue (e.g., as in the late conditions of Experiments 1 and 3), more information corresponding to an earlier representation (i.e., vertical orientation) is accumulated because of an expedited onset or accelerated rate in the accumulation process, resulting in weaker repulsion. As such, we can map the models that normally explain the task performance onto phenomenological appearance (Fig. 1; see also Denison et al., 2022).

### Potential influence of inducer duration

To ensure that the inducer and cues were uninformative about the target-onset conditions, each trial followed the same presentation protocol regarding the time courses of these items until the delivery of the target, regardless of the condition. In Experiment 1, however, fixing the protocol inevitably caused the inducer duration to vary across conditions – 1,400, 1,600, and 1,800 ms in the early, on-time, and late conditions, respectively. As such, the inducer onset per se technically served as one of the temporal cues. At the same time, however, it also provided a source of variability within vision-specific mechanisms other than the variability of temporal predictability. Several points concerning this potential issue are worth mentioning. First, it is unlikely that our participants were aware of the abovementioned differences in inducer duration between conditions, because the differences were much smaller than the typical discrimination threshold for the duration of a static visual stimulus (e.g., Grondin, 1993). Second, image fading via adaptation during inducer duration is also unlikely because the inducer had a high luminance contrast (99%) on a bright background (31 cd/m^2^). Third, attenuation within the orientation contrast mechanism over time is also unlikely because the orientation repulsion size generally increases with increasing inducer duration (Nakamura & Murakami, 2022), contrary to the present data.

Nonetheless, we verified whether inducer duration per se exerted any effect by analyzing the data from Experiment 3, in which the inducer–cue SOA was randomly chosen (see the double arrow in Fig. 10A). We median-split the data based on the length of the inducer–cue SOA for each of the early, middle, and late conditions, and separately estimated the orientation repulsion size for each of the six combinations (shorter- and longer-than-median inducer–cue SOAs × three foreperiods). A two-way rm-ANOVA revealed no significant main effect of the inducer–cue SOA (*F*(1, 10) = 0.05, *p* = .830, ${\eta }_{p}^{2}$ = .005), while the main effect of the foreperiod remained significant (*F*(1.6, 15.9) = 20.41, *p* < .001, ${\eta }_{p}^{2}$ = .671). The interaction was not significant either (*F*(1.6, 16.1) = 2.43, *p* = .127, ${\eta }_{p}^{2}$ = .196). Therefore, we conclude that inducer duration per se did not effectively contribute to the reduction in orientation repulsion.

### Temporal expectation and other related concepts

In natural situations, it is often beneficial to allocate limited resources to the moments with the highest probabilities of event occurrence; that is, “temporal expectation” is likely to covary with “temporal attention.” Even in laboratory settings where a single “target” is always task-relevant, temporal expectation regarding the target onset also directs temporal attention to the expected target onset time. Our study is no exception; instructing and briefly training participants to form internalized predictions about the statistical structure of the world may allow them to allocate their attention to specific moments. Thus, although they are suggested to convey different concepts (e.g., Duyar et al., 2023; Todorovic et al., 2015; see also Summerfield & Egner, 2009), these terms may be interchangeable for descriptive purposes. Nonetheless, since the common independent variable in our experiments was the probability of stimulus onset as a function of time, we have tentatively used the more neutral term “temporal expectation,” without excluding the possibility that temporal expectation we manipulated actually altered repulsion via temporal attention.

Furthermore, regardless of the involvement of temporal attention, our findings are consistent with the established roles of temporal predictability in time-sensitive behaviors (e.g., classical conditioning, sensorimotor learning, and temporal recalibration). Even if the reduction of orientation repulsion shares a common mechanism with one of these various phenomena, our main argument – that temporal expectation results in earlier access to a premature representation corresponding to an altered visual appearance – is not undermined. In addition, it is unlikely that the observed effect of expectation and/or attention in the temporal domain can be generalized to the spatial domain, because a previous study found no evidence that manipulations of predictability regarding target location influence orientation repulsion in a similar stimulus configuration (Nakamura & Murakami, 2023).

### Repulsive bias in orientation

Repulsive bias in the orientation domain arises not only from the spatial context but also from the temporal context. In repulsive serial dependence, the orientation estimation of the current stimulus is biased away from that in the previous trial (e.g., Fritsche et al., 2017; Pascucci et al., 2023), reminiscent of the classical tilt aftereffect (e.g., Gibson & Radner, 1937). Even in the absence of any spatial or temporal context, the perception of oblique orientations can be biased away from cardinal orientations (so-called cardinal bias; see, e.g., Girshick et al., 2011; Tomassini et al., 2010; Wei & Stocker, 2015).

Our study was not designed to examine serial dependence or cardinal bias, as the target orientation was always constrained to lie within a narrow range (± 6° around the subjective verticality; cf., Bae & Luck, 2019, 2020). We assume that any serial dependence, if present, would have been canceled out because of the random-order selection of the target orientation in each trial. If anything that looked like cardinal bias occurred in the context of the method of constant stimuli that we employed, the bias would symmetrically affect both positive and negative orientations with respect to the subjective verticality, resulting in a change in the slope of the psychometric function (see *Discussion* in Patten & Clifford, 2015). However, we found no evidence of changes in discrimination thresholds across conditions in any of our experiments. Therefore, it is unlikely that serial dependence or cardinal bias contaminated the observed effects of temporal expectations on orientation repulsion.

Assuming that all of these repulsive biases reflect the fundamental functionality of the visual system in effectively detecting spatiotemporal changes in the environment (Fritshce et al., 2017), it becomes intriguing to investigate the interactions among these biases and how they might be modulated by temporal expectations. For instance, Bae (2024) demonstrated that repulsive serial bias occurs only when the direction predicted by the cardinal bias is congruent, not when it is incongruent. This interaction suggests that the processing stages underlying repulsive serial bias and cardinal bias overlap, at least to some extent. Regarding susceptibility to foreperiod manipulation (cf. our Experiment 3), repulsive serial dependence decreases and even reverses its direction as the delay between stimuli and response increases (Bliss et al., 2017). When orientation is continuously reported through the observer’s action with a computer mouse, a similar temporal evolution of bias occurs in its trajectory (Chen & Bae, 2024). As such, investigating these repulsive orientation biases within a unified paradigm, particularly focusing on temporal dynamics, will enhance our understanding of the locus and dynamic mechanisms underlying orientation perception in general.

## Conclusions

We observed a decrease in orientation repulsion when the target appeared at a more predictable moment. The findings indicate that developing temporal expectations not only enhances behavioral performance but also leads to a change in phenomenological experience. We suggest that both outcomes can be consistently explained by the reduction of perceptual latency associated with temporal expectations, which enables a premature representation to become consciously accessible even before contextual modulation is fully completed.

## Data Availability

All data are publicly available from the Open Science Framework (OSF) at: https://osf.io/42fh7/ The study design and analysis plan were not pre-registered.

## Code availability

The code used in this study is available from the corresponding author upon request.
